# Oscillatory shear stress-driven endothelial-to-mesenchymal transition: a critical mechanical signal transduction mechanism in atherosclerosis progression

**DOI:** 10.1038/s41420-026-03000-6

**Published:** 2026-03-10

**Authors:** Jie Li, Wenchao Xu, Jie Ju, Min Liu, Wenxu Wang, Min Cheng, Xiaoyun Zhang, Xiaodong Cui, Hao Chen

**Affiliations:** 1 Department of Physiology & Pathophysiology, School of Basic Medical Sciences, Shandong Second Medical University, Weifang, Shandong PR China; 2 Weifang Key Laboratory of Basic Research on Chronic Diseases and Stem Cell Therapy, School of Basic Medical Sciences, Shandong Second Medical University, Weifang, Shandong PR China; 3 Department of Physiology, School of Basic Medical Sciences, Shandong Second Medical University, Weifang, Shandong PR China; 4 Department of neurology, Sunshine Union Hospital, Weifang, Shandong PR China

**Keywords:** Atherosclerosis, Ion channel signalling, Atherosclerosis

## Abstract

Atherosclerosis constitutes the primary pathological basis for cardiovascular diseases. It most commonly develops at the branching and curved regions of blood vessels. The disturbed blood flow in these regions can generate oscillatory shear stress (OSS). Endothelial cells exposed to OSS progressively undergo a transformation into mesenchymal cells, a process known as endothelial-to-mesenchymal transition (EndMT). EndMT is a critical event in the development of atherosclerosis. OSS promotes the occurrence of EndMT through multiple pathways. This paper provides a comprehensive analysis of the phenomena and mechanisms of OSS-induced EndMT, offering theoretical insights into the pathogenic mechanisms of atherosclerosis and corresponding therapeutic strategies.

## Facts

- Oscillatory shear stress is a key factor in inducing endothelial-to-mesenchymal transition (EndMT).
- Mechanical sensors convert physical mechanical signals into chemical signals that can regulate endothelial cell function.
- EndMT causes alterations in both the morphology and function of endothelial cells, serving as an initiating factor that triggers the onset of atherosclerosis.

## Open questions

- Why is atherosclerosis more likely to occur at the curved and branching parts of blood vessels?
- What role do endothelial cells play in atherosclerosis?
- How do physical mechanical signals affect the function of endothelial cells?
- Is inhibiting EndMT a new direction for treating atherosclerosis?

## Introduction

Atherosclerotic cardiovascular disease is a prevalent and serious cardiovascular issue that manifests in various forms, including ischemic heart disease, ischemic stroke, and peripheral artery disease. Atherosclerosis is particularly common at the branching and curved regions of blood vessels, where disturbed blood flow can damage the endothelial layer [1]. Endothelial cells (ECs) form a monolayer of tightly connected cells covering the surface of the vascular endothelium, serving as an interface between blood and the vascular wall [2]. ECs injury is a triggering factor in the onset and progression of atherosclerosis [3]. Under disturbed flow conditions, mechanical sensors on ECs convert physical shear stress into biochemical signals, thereby ultimately modulating cellular functions [4, 5]. Endothelial-to-mesenchymal transition (EndMT) is an adaptive change that occurs in ECs under the influence of oscillatory shear stress (OSS) generated by disturbed flow [6] and is a critical event in the progression of atherosclerosis [7]. This paper aims to provide a comprehensive review of the pivotal role that OSS plays in EndMT during the development of atherosclerosis, along with the underlying molecular mechanisms, thereby offering new insights into the pathogenesis of atherosclerosis.

## Hemodynamics and atherosclerosis

The vascular wall primarily experiences two critical mechanical forces: circumferential tension and shear stress [8]. The cardiac cyclical motion drives blood flow, resulting in periodic circumferential expansion and stretching of the vessel wall. This tensile force is perpendicular to the vessel wall and is also called circumferential tension. Smooth muscle cells arranged circumferentially in the media respond to this circumferential tension [9]. Shear stress specifically refers to the frictional force exerted by flowing blood on the surface per unit area of the vascular wall, mainly acting on the ECs arranged longitudinally in the intima of the arterial wall [10].

The flow patterns of blood exhibit significant differences based on the geometry of the blood vessels. In straight and unbranched regions, blood flow is relatively stable, while in branched and curved regions, the blood flow is disturbed [11]. Shear stress can be further classified into laminar shear stress (LSS) and oscillatory shear stress (OSS) based on its flow characteristics. LSS ( > 12 dyn/cm²) mainly occurs in areas with stable blood flow, such as the abdominal aorta. At this time, the blood flow is smooth and continuous, without obvious disturbance. LSS can inhibit the expression of adhesion factors in ECs [12], suppress inflammatory responses [13], and ultimately inhibit the formation of atherosclerosis [14]. In contrast, OSS ( ± 4 dyn/cm²) primarily exists in areas of vascular branching or curvature. In these regions, the direction of blood flow changes, creating turbulence. Unlike LSS, OSS promotes ECs proliferation, inflammatory response [15, 16], leukocyte adhesion, oxidative stress [17] and other pathological processes, thus accelerating the formation of atherosclerosis.

## OSS drives EndMT to occur

**Fig. 1:**
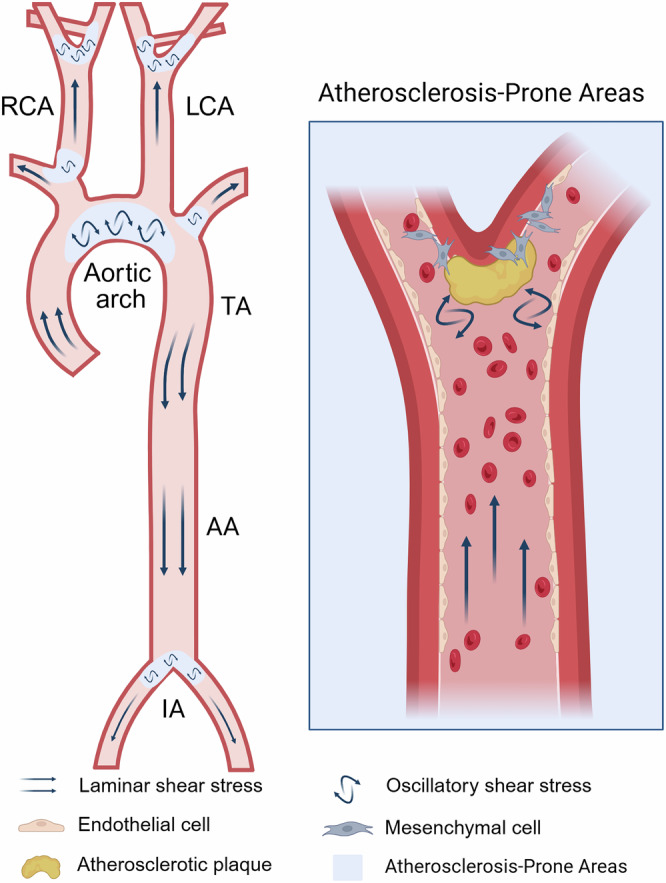
Schematic diagram of vascular distribution showing OSS-induced EndMT accelerating atherosclerosis. In straight, unbranched regions of blood vessels, stable blood flow results in the formation of LSS. In branched and curved regions, disturbed blood flow leads to the generation of OSS. ECs exposed to OSS undergo transformation into mesenchymal cells, thereby accelerating the progression of atherosclerosis. AA abdominal aorta, TA thoracic aorta, IA iliac artery. (Created with BioRender.com).

In regions of disturbed blood flow, OSS damages ECs, causing them to gradually lose their endothelial characteristics and adopt a mesenchymal cell phenotype. This process is referred to as EndMT [18] (Fig. 1).

### EndMT is commonly found in human atherosclerotic plaques

Based on single-cell transcriptome sequencing analysis of plaque tissue from patients with atherosclerosis, ECs were divided into four subclasses E.0-E.3. The E.0, E.1, and E.2 subtypes exhibited classical endothelial markers CD34 and CD31, as well as the vascular endothelial marker TIE1. The E.3 subtype expressed smooth muscle cell markers: actin, aortic smooth muscle (ACTA2), neurogenic locus notch homolog protein 3 (NOTCH3), and myosin-11 (MYH11). Cluster analysis and functional enrichment analysis indicated that these samples underwent EndMT [19]. Studies showed that the activation of EndMT was closely related to the severity and stability of atherosclerotic plaques. Abnormal increases of smooth muscle cell markers (NOTCH3 and transgelin) and mesenchymal cell markers (collagen 1 and fibronectin) were simultaneously detected in the coronary artery ECs of patients with atherosclerosis. Additionally, the expression level of the mesenchymal cell markers was positively correlated with the ratio of fiber cap thickness to lipid core diameter. The ratio of fiber cap thickness to lipid core diameter is an important indicator for evaluating plaque stability. This suggested that the activation of EndMT is directly related to the severity of atherosclerosis [20]. By comparing stable plaques with unstable plaques, it was found that the ratios of mesenchymal/endothelial markers [fibroblast activation protein alpha/von Willebrand factor (FAP/vWF), ferroptosis suppressor protein 1/von Willebrand factor (FSP-1/vWF)] were significantly increased in unstable plaques. The proportion of FAP/vWF double-positive cells in ruptured plaques was higher [21, 22]. In human coronary atherosclerotic plaques, the fibrous cap in plaque rupture and intraplaque hemorrhage regions is thinner than that in fibroatheroma regions, indicating higher plaque instability. ECs co-expressing endothelial markers (such as CD31) and mesenchymal markers (such as FSP-1) indicate ongoing EndMT. A greater number of cells undergoing EndMT (CD31/FSP-1 double-positive cells) are present in plaque rupture and intraplaque hemorrhage regions [23]. Snail1 serves as a key transcription factor for EndMT initiation. Snail1 expression was not detected in stable human atherosclerotic plaques, whereas its expression was significantly elevated in unstable plaques [24]. These findings suggest that EndMT is closely associated with atherosclerotic plaque instability. Specifically, EndMT activation not only promotes the progression of plaques to more severe pathological stages but also enhances plaque instability by impairing the structural integrity of the fibrous cap.

### Disturbed blood flow induces EndMT and atherosclerosis in animal models

**Fig. 2:**
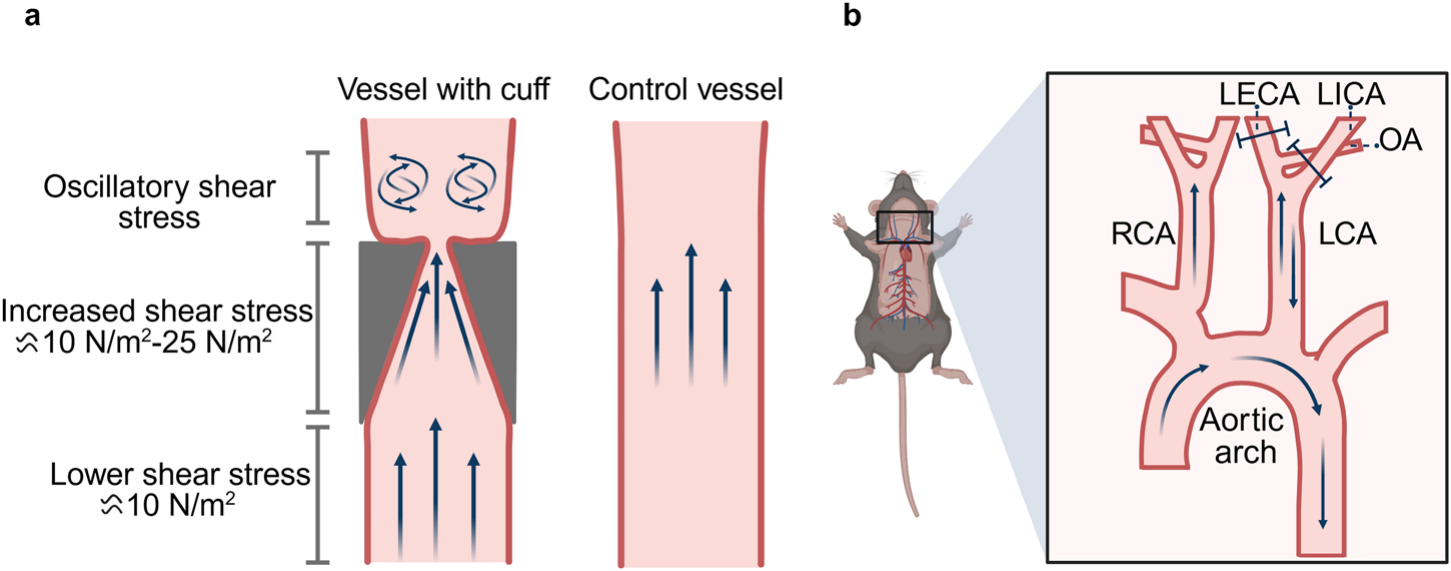
Schematic diagram of disturbed blood flow inducing EndMT and atherosclerosis in animal models. **a** Schematic diagram of the constrictive carotid artery cuff model. The image on the left shows the hemodynamic changes of the carotid artery in mice after cuff implantation, with OSS forming above the cuff. The image on the right shows the straight segment of the carotid artery in mice without cuff implantation, with blood flow maintained in a stable laminar state [28]. **b** Schematic diagram of cervical ligation of the left common carotid artery. By ligating the three main caudal branches of the left common carotid artery in mice while keeping only the superior thyroid artery intact, local hemodynamic disorders are caused, thereby constructing an accelerated model of atherosclerosis. LECA left external carotid artery, LICA left internal carotid artery, OA occipital artery. (Created with BioRender.com).

The constrictive carotid artery cuff model is usually made by fabricating a cuff with a tubular structure made of biocompatible materials (such as silicone, polytetrafluoroethylene) and then placing the cuff on the exposed mice left or right carotid artery [25, 26] (Fig. 2a). By adjusting the size and tightness of the cuff, different degrees of carotid artery stenosis or compression can be simulated [27]. In the constrictive carotid cuff mouse model, the shear stress in the region beneath the cuff was lowest (≈10 N/m²). Due to the conical shape of the cuff, the shear stress was highest in the cuffed region (≈25 N/m²). OSS in the area above the cuff decreased to approximately 14 N/m² [28]. Studies showed that the left carotid artery of mice maintained stable morphological characteristics as the control group, with the intima-media thickness value of the carotid artery ranging from 10.8 to 19.1 µm. In the right carotid artery of mice, as a constrictive carotid artery cuff model, significant thickening of the carotid intima-media thickness (24.7-69.2 µm) and severe endothelial injury were observed in the tissue samples at the late stage (10 weeks and 12 weeks after surgery) [29].

The left carotid artery (LCA) ligation surgery also known as partial carotid ligation (PCL) is a method used to induce atherosclerosis by altering the blood flow conditions in the carotid artery (Fig. 2b). Ligate three of the four caudal branches of the left common carotid artery in mice, namely the left external carotid artery, the left internal carotid artery and the left occipital artery. Meanwhile, the integrity of the superior thyroid artery is maintained to cause local blood flow disorders and changes in shear stress, thereby accelerating the formation of atherosclerosis. The right common carotid artery (RCA) is untreated and serves as a control [26]. Researchers found that PCL in wild-type C57BL/6 mice (WT mice) induces intimal hyperplasia characterized by the proliferation and migration of vascular smooth muscle cells, but fails to form typical atherosclerotic plaques [30–32]. In the WT mouse PCL model, hemodynamic changes were observed on postoperative days 1 and 7. Specifically, the LCA exhibited a reduction in systolic blood flow velocity and a 90% decrease in blood volume. Additionally, the direction of diastolic blood flow in the LCA was reversed, forming typical disturbed flow. In contrast, no significant change in blood volume was detected in the RCA [30]. Two weeks after surgery, the LCA lumen diameter decreased by approximately 13% with unchanged vascular wall thickness [33], and the elastic lamina became thinned and fragmented. Only an increase in lumen area was noted in the RCA. At 4 weeks postoperatively, the LCA media thickened by 50%, while the RCA media showed only mild thickening [34]. Further studies revealed that PCL combined with a high-fat diet for 3 months did not induce obvious atherosclerotic plaques in WT mice, whereas ApoE^–/–^ mice rapidly developed atherosclerosis via disturbed flow within 2 weeks of PCL plus high-fat diet. This indicates that the use of gene knockout models (e.g., ApoE^–/–^ mice) is critical for inducing atherosclerosis. ApoE^–/–^ mice fed only a high-fat diet without PCL require 3 months or longer to develop significant atherosclerotic plaques. This confirms that PCL synergizes with hypercholesterolemia through hemodynamic changes, significantly accelerating the progression of atherosclerosis [35]. The rapid establishment of atherosclerosis models by combining PCL with a high-fat diet in ApoE^–/–^ mice has been widely applied in research.

Following PCL in ApoE^–/–^ mice, OSS was observed in the LCA, which was a characteristic of disturbed blood flow [36]. Compared to the RCA, the expression of atherosclerosis-promoting genes bone morphogenetic protein 4 (BMP4), intercellular adhesion molecule 1 (ICAM-1), and vascular cell adhesion molecule 1 (VCAM-1) was significantly upregulated in the LCA. On the 7th day of modeling, the relaxation response of LCA to acetylcholine was significantly inhibited. However, the degree of relaxation of the endothelium-independent vasodilator sodium nitroprusside by LCA and RCA was almost the same. This indicated that the disturbed blood flow impaired endothelial function within 7 days [30]. When analyzing the LCA tissue sections of mice after PCL, it was found that a large number of cells simultaneously expressed the endothelial marker CD31 as well as the EndMT markers ACTA2, NOTCH3, and fibronectin [37]. Additionally, compared to the RCA, there was a significant reduction in the expression levels of endothelial markers [CD31, CD34, vWF, and cadherin-5 (CDH5)]. The expression of mesenchymal markers (transgelin, CD44, vimentin, and ACTA2) significantly increased. Meanwhile, obvious pathological changes occurred in the LCA region, including the infiltration of monocytes, thickening of the intima, lipid deposition, and the formation of circular plaques. In contrast, no significant plaque was observed in the RCA region [38]. These findings indicated that the blood flow disturbances induced by PCL not only promoted the occurrence of EndMT but also accelerated the formation of atherosclerotic plaques.

### OSS induces ECs to undergo EndMT and accelerates atherosclerosis

Cell polarity refers to the uneven spatial distribution of cytoplasmic components and is an important basis for the normal functioning of cells. Studies found that OSS damaged the planar cell polarity of vascular ECs. The specific manifestations were the decrease in cell elongation rate and nuclear ellipticity, as well as the increase in cell density [39]. During the trans-differentiation process of ECs, the originally pebble-shaped and closely arranged ECs transform into spindle-shaped mesenchymal cell morphology [40, 41]. Immunofluorescence stain of endothelial junction adhesion protein CDH5 indicated that under OSS, the adhesion connection between ECs was interrupted [38]. The intercellular contact and adhesion to the basement membrane were lost [42]. The above changes indicated that under OSS, the cytoskeleton of ECs underwent rearrangement. The structure transitioned from its original compact configuration, which was abundant in microfilaments, to a mesenchymal cytoskeleton characterized by an increased presence of actin stress fibers [43].

**Fig. 3:**
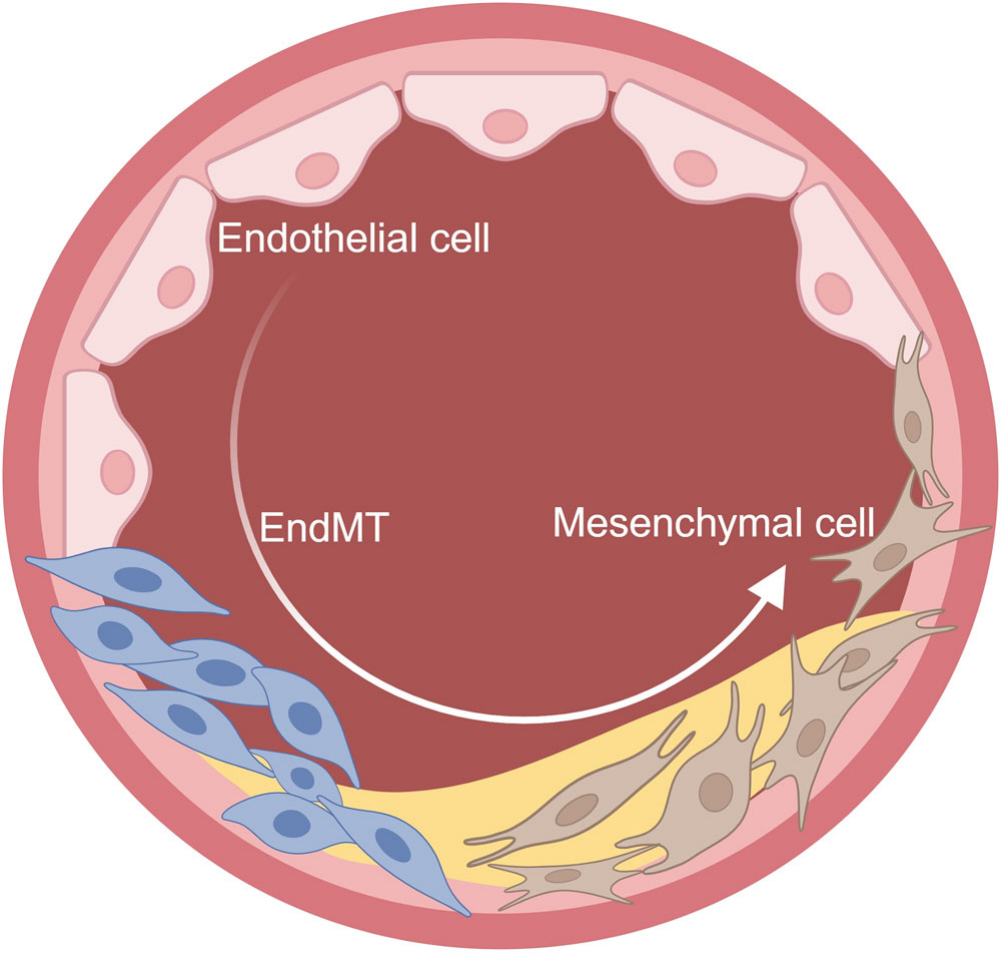
The conversion process of ECs to mesenchymal cells. ECs first transition to an EndMT intermediate state before evolving into mesenchymal cells. (Created with BioRender.com).

EndMT is a complex and dynamic process. During this process, the expression of the original markers in ECs gradually weakens, while the expression of markers in mesenchymal cells significantly increases [41, 44]. ECs undergoing EndMT simultaneously exhibit markers of both cell types, reflecting their transitional state characteristics [45], which is termed partial EndMT [46] (Table 1). The trajectory plot clearly showed the transformation process of ECs labeled by endothelial maker CD31 and mesenchymal cell marker myosin light chain 9 (MYL9). Pseudotime analysis revealed that ECs, EndMT, and mesenchymal cells distinctly clustered and overlapped on the timeline, showing a typical distribution of different cell types. This suggested that ECs transformed into the EndMT state at first and finally evolved into mesenchymal cells [47] (Fig. 3). During partial EndMT, ECs may only temporarily adjust their phenotype to accommodate specific physiological or pathological environments [48]. Under certain conditions, EndMT can be reversed. This partial EndMT process enables ECs to retain residual endothelial functions, such as tube formation, while simultaneously acquiring mesenchymal characteristics, particularly enhanced migratory capacity [49]. In contrast, complete EndMT refers to the full transformation of ECs into mesenchymal cells. This process is not merely a transient adjustment of cell phenotype but involves bigger changes in cellular functions, morphology, and gene expression. In complete EndMT, ECs downregulate endothelial markers (CD31, vWF, CD34) and upregulate mesenchymal markers (ACTA2, transgelin, NOTCH3). Studies found that the complete EndMT existed in atherosclerotic plaques. During the 8th and 30th weeks of the atherosclerotic mouse model, approximately 32.5% and 45.5% of the mesenchymal cells in atherosclerotic plaques originated from ECs, respectively [22].

**Table 1:** Summary of endothelial and mesenchymal cell markers.

Endothelial cell maker		Mesenchymal cell maker			
Protein name	Gene name	Protein name	Gene name	Protein name	Gene name
CD31	PECAM1 [19]	Transgelin/SM22α [38]	TAGLN [20]	Myocardin	Myocd [47]
CD34	CD34 [19]	α-SMA [154]	ACTA2 [19]	Fibronectin [20]	FN1 [37]
VE-Cadherin [154]	CDH5 [38]	Myosin-11	MYH11 [19]	N-cadherin [20]	CDH2 [95]
Claudin-5	Cldn5 [47]	FSP-1 [22]	AIFM2	Vimentin	Vim [38]
CD9 [151]	CD9	CD44 molecule	CD44 [38]	Tropomyosin 2	TPM2 [151]
von Willebrand factor	vWF [22]	Calponin-1 [154]	CNN1 [47]	Plasminogen activator inhibitor 1	Serpine1 [48]
Cytokine-like protein 1	CYTL1 [47]	Integrin alpha-9	ITGA9 [47]	Collagen alpha-1(I) chain	COL1A1 [47]
Intercellular adhesion molecule 1	ICAM1 [66]	Serine protease 23	PRSS23 [151]	Collagen alpha-1(III) chain	COL3A1 [48]
Cadherin EGF LAG seven-pass G-type receptor 3	EGFL1 [47]	Myotonin-protein kinase	Dmpk [47]	Zinc finger E-box-binding homeobox 1	ZEB1 [124]
Vascular endothelial growth factor receptor 2	VEGFR2 [95]	Zinc finger protein SNAI2	Slug/Snai2 [20]	Zinc finger E-box binding homeobox 2	ZEB2 [20]
Transcriptional regulator ERG	ERG [135]	Snail family transcriptional repressor 1	Snai1 [20]	Fibroblast activation protein alpha	FAP [22]
Tyrosine-protein kinase receptor Tie-1	TIE1 [19]	Neurogenic locus notch homolog protein 3	NOTCH3 [19]	CTGF (connective tissue growth factor)	CCN2 [95]
Angiopoietin-1 receptor	TIE2 [95]	Platelet-derived growth factor receptor alpha	Pdgfra [48]	Vascular cell adhesion protein 1	VCAM1 [95]
Secreted frizzled-related protein 1	SFRP1 [151]	Myosin regulatory light polypeptide 9	MYL9 [47]	Metalloproteinase inhibitor 1	TIMP1 [151]

*PECAM-1* platelet endothelial cell adhesion molecule-1, *VE-cadherin* vascular endothelial cadherin, *CDH5* cadherin-5, *a-SMA* alpha smooth muscle actin, *ACTA2* actin, aortic smooth muscle, *FSP-1* ferroptosis suppressor protein 1, *CDH2* cadherin-2, *N-cadherin* neural cadherin.

During the process of EndMT, the morphological transformation not only alters the structure of ECs but also significantly changes their functions. The most prominent change is that the barrier function of ECs is severely disrupted. Originally, ECs have tight junctions covering the inner surface of the blood vessel, serving as a barrier between the blood and the vascular wall tissue [2]. However, during the process of EndMT, the expression levels of adhesion molecules such as CD34, CDH5, and Claudin-5 in ECs are downregulated, resulting in a reduction in intercellular adhesion connections [50]. At the same time, the cytoskeleton undergoes rearrangement, further weakening the barrier function of ECs. This makes lipids and inflammatory cells in the blood more likely to penetrate the vascular wall, promoting the early lesions of atherosclerosis [51]. During trans-differentiation, endothelial-derived mesenchymal cells exhibit enhanced migratory ability, proliferation, and contractility [52]. Changes in cell morphology and enhanced expression of fibroblast actin, vimentin and FSP-1 resulted in increased cell migration ability [45, 53]. Studies showed that when human umbilical vein endothelial cells (HUVECs) were exposed to OSS, the proliferation ability was significantly enhanced. The permeability to macromolecules such as albumin was also increased. This phenomenon was closely related to the increased expression of the transcription factor Snail during EndMT [54]. The expression of alpha smooth muscle actin (α-SMA), MYH11, smooth muscle 22 alpha (SM22α), and other proteins involved in the contraction process of mesenchymal cells was also significantly increased, which promoted the contraction ability of transdifferentiated cells [55].

In summary, the reduction of ECs adhesion junctions, remodeling of the cytoskeleton, up-regulation of mesenchymal markers, and changes in cell morphology during EndMT together lead to significant changes in ECs function. Functional changes not only affect the permeability and stability of blood vessels but also promote the occurrence and development of atherosclerosis. The transformed mesenchymal cells have the ability to synthesize and secrete extracellular matrix (collagen 1α, fibronectin). However, excessive production of the matrix can lead to tissue fibrosis [56], subsequently resulting in tissue dysfunction [57]. Endothelial-derived mesenchymal cells also produce metalloproteinases, which enhance the formation and increase the instability of atherosclerotic plaques [23]. It has been shown that the degree of EndMT was strongly associated with an unstable plaque phenotype, as indicated by a thinning of the fibrous cap of the plaque and a predisposition to plaque rupture [53]. At the same time, endothelial injury triggers an inflammatory response within the vessel wall that recruits inflammatory cells such as macrophages to migrate to the damaged area. Macrophages or proliferating smooth muscle cells form foam cells after phagocytosing a large amount of oxidized low-density lipoprotein. These foam cells gradually accumulate within the arterial wall, forming lipid streaks and even lipid plaques. This is a typical pathological feature of atherosclerosis [58].

## Mechanical sensors on ECs respond to shear stress

**Fig. 4:**
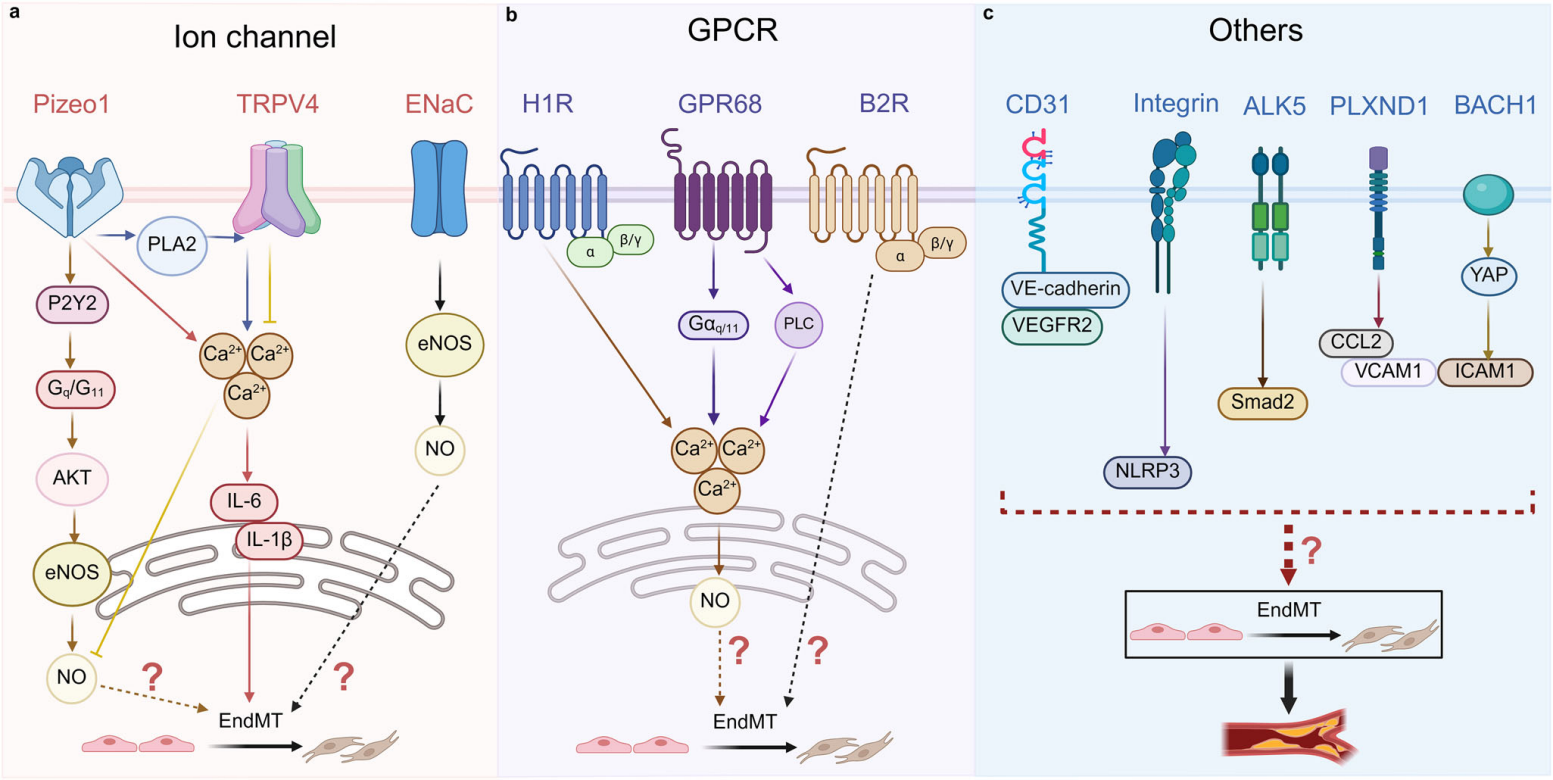
Mechanical sensors mediating shear stress in ECs. They mainly include ion channel class: Piezo1, TRPV4, ENaC (**a**); GPCR class: H1R, GPR68, B2R (**b**); others: CD31, Integrin, ALK5, PLXND1, BACH1 (**c**). (Created with BioRender.com).

The differences in blood flow patterns are mainly perceived by mechanical sensors on ECs. Mechanical sensors convert mechanical force stimuli into biochemical signals, enabling cells to respond to external or internal mechanical changes and ultimately regulating cell and organ functions. The above process is called mechanical conduction [59]. Shear stress strongly regulates the structure, function, transcriptome, epigenome, and metabolic changes of ECs through mechanical sensors and mechanical transduction pathways. This is particularly crucial for the homeostasis and normal physiological functions of vascular ECs [26] (Table 2) (Fig. 4).

**Table 2:** Mechanical sensor summary table.

Categories	Mechanical sensors	Appropriate stimulus	Mechanism	Effects
Ion Channel	Piezo1	Shear stress	(1) Piezo1-PLA2-TRPV4-Ca^2+^ [62] (2) Piezo1-Ca^2+^-CD31[63]	Regulate the structure of endothelial cells
			Piezo1-P2Y_2_-G_q_/G_11_-AKT-eNOS-NO [64]	Regulate vascular tension and blood pressure
			Piezo1-Ca^2+^-sAC-IP3R2-Ca^2+^ [65]	Regulate the morphology of endothelial cells and vascular function
		Disordered blood flow	Piezo1-Ca²⁺-IL-6、IL-1β-EndMT [66]	Regulate the occurrence of vascular inflammation and atherosclerosis
	TRPV4	Shear stress	TRPV4-Ca²⁺-NO [68, 69]	Causes vasodilation
			Formation of the TRPV4-TRPC1-TRPP2 complex [72]	Regulate intracellular Ca²⁺ concentration and cell function
	ENaC	Shear stress	ENaC-eNOS-NO [83]	Regulate endothelial function and vascular tension
GPCR	H1R	Mechanical stimulation	H1R-G protein-Ca²⁺-NO [85]	Causes vasodilation
	GPR68	Shear stress	(1) GPR68-Gα_q/11_-Ca^2+^ (2) GPR68-PLC-Ca^2+^ [87]	Causing vasodilation and remodeling
	B2R	Fluid shear force, low osmotic stress	B2R conformational change-G protein [90]	Responding to mechanical forces’ stimulation
Others	CD31	Fluid shear stress	CD31, VE-cadherin, and VEGFR2 mechanical sensing complex [91]	Reshape the structure of cell connections and affect the migration ability of endothelial cells
	Integrin	Oscillatory shear stress	Move the integrin α5 [98, 99]	Regulate endothelial function, promote inflammation and atherosclerosis
	ALK5	Oscillatory shear stress	OSS-ALK5-Shc-Smad2 [37]	Regulation of EndMT and Atherosclerosis
	PLXND1	Shear stress	Formation of the PLXND1/NRP1/VEGFR2 mechanical complex [106]	Regulate endothelial function, promote inflammation atherosclerosis
	BACH1	Oscillatory shear stress	BACH1-YAP [108]	Inducing vascular inflammation and the formation of atherosclerosis

*Piezo1* Piezo-type mechanosensitive ion channel component 1, *PLA2* phospholipase A2, *TRPV4* transient receptor potential vanilloid 4, *P2Y*_2_ P2Y purinoceptor 2, *AKT* protein kinase B, *eNOS* endothelial nitric oxide synthase, *sAC* soluble adenylyl cyclase, *IP3R2* inositol 1,4,5-trisphosphate receptor type 2, *TGF-β* transforming growth factor-beta, *EMT* epithelial-mesenchymal transition, *ENaC* epithelial sodium channel, *GPCR* G protein-coupled receptor, *H1R* H1 receptor, *GPR68* G protein-coupled receptor 68, *PLC* phospholipase C, *B2R* Bradykinin 2 receptor, *VEGFR2* vascular endothelial growth factor receptor 2, *ALK5* activin receptor-like kinase 5, *Shc* src homology and collagen, *NRP1* neuropilin-1, *BACH1* BTB and CNC homology 1.

### Ion channels

#### Piezo1

Piezo-type mechanosensitive ion channel component 1 (Piezo1) is a transmembrane protein that effectively senses shear stress through a unique trimer propeller-like structure and converts this mechanical signal into intracellular electrochemical signals [60]. Piezo1 is expressed in the vascular ECs. Embryos deficient in Piezo1 died due to vascular remodeling disorders in the second trimester of pregnancy. When Piezo1 was depleted in ECs, ECs exhibited defects in stress fiber formation and cell orientation under shear stress [61].

As a key mechanosensitive cation channel on ECs, Piezo1 forms a complex signal network with other mechanical sensors [transient receptor potential vanilloid 4 (TRPV4), CD31]to regulate the function and adaptive response of ECs. Piezo1 stimulated the opening of TRPV4 by activating phospholipase A2 (PLA2), resulting in a continuous increase in intracellular Ca^2+^. This was an important cause of shear stress-mediated adhesion connection disruption and actin remodeling [62]. Furthermore, Piezo1 and CD31 formed in situ overlaps at the junctions between ECs. CD31, as a guiding molecule, assisted Piezo1 in locating the junctions between ECs. CD31 and CDH5 further collaborated with Piezo1 to jointly respond to shear stress. This process relies on the influx of Ca^2+^ induced by mechanical force, thereby promoting the remodeling of the structure at the cell junctions and the cytoskeleton [63].

Piezo1 plays an important role in maintaining the normal physiological activities of blood vessels. Shear stress first triggered Piezo1-mediated adenosine triphosphate (ATP) release, activated the P2Y purinoceptor 2 (P2Y_2_) purinergic receptor and G_q_/G_11_, and further activated protein kinase B (AKT). The activation of AKT further promotes the activation of endothelial nitric oxide synthase (eNOS), facilitating the generation and release of nitric oxide (NO), thereby regulating vascular tension and blood pressure [64]. The Piezo1-soluble adenylyl cyclase (sAC)-inositol 1,4,5-trisphosphate receptor type 2 (IP3R2) pathway is one of the important mechanisms for ECs' morphological adaptation and vascular function maintenance. After the shear stress activates Piezo1, it rapidly mobilizes intracellular Ca^2+^ into the endoplasmic reticulum, and then rapidly releases Ca^2+^ in the endoplasmic reticulum through the cyclic adenosine monophosphate (cAMP)-sensitive IP3R2 channel. The activation of IP3R2 amplified calcium signals, thereby inducing an adaptive response of ECs to shear stress. Meanwhile, this process is further facilitated by sAC-mediated cAMP production, ensuring that ECs can accurately sense and respond to changes in shear stress [65].

Recent studies found that the Piezo1 channel mediated EndMT induced by disordered blood flow. The disordered blood flow activated Piezo1 channels and triggered a large amount of Ca²⁺ influx, leading to the release of inflammatory factors [interleukin-6 (IL-6) and interleukin-1 β (IL-1β)]. The release of inflammatory factors further intensified the inflammatory response of the vascular wall, providing conditions for the occurrence and development of atherosclerosis. In addition, the activation of the Piezo1 channel also promoted EndMT. During this process, the expression of the mesenchymal cell marker α-SMA was up-regulated, and the endothelial marker ICAM1 was down-regulated. After knocking out the Piezo1 channel in the mouse after PCL, although the blood flow disorder still existed, the influx of Ca²⁺ was significantly inhibited. This change led to a reduction in the release of downstream IL-6 and IL-1β, which reduced the extent of the vascular inflammatory response. This study confirmed that the Ca²⁺ influx caused by the activation of Piezo1 due to disturbed blood flow is correlated with EndMT. However, it did not clarify whether there is a causal relationship between the two. Meanwhile, Piezo1 inhibitor GsMTx4 significantly inhibited the expression of EndMT markers caused by Piezo1 activation [66]. The above results confirmed that Piezo1 played a key mechanical conduction role in mediating vascular inflammation and EndMT induced by blood flow disorders on ECs.

#### TRPV4

TRPV4 belongs to the TRP ion channel family. Under the stimulation of shear stress, the distribution and conformation of TRPV4 molecules changed, forming smaller clusters and being confined to the basement membrane [67]. This is closely related to the mechanical conduction effect of TRPV4 on ECs. In response to shear stress, TRPV4 mediated Ca²⁺ signals to promote vasodilation. The activation of Ca²⁺ signaling triggered the release of NO and endothelial relaxation factors, eventually leading to vasodilation [68, 69]. When the function of TRPV4 is impaired, the dilation function of blood vessels under shear stress is affected [70]. In response to this phenomenon, existing studies revealed the potential mechanism by which TRPV4 promoted vasodilation. There is a Ca²⁺ influx of a single TRPV4 cation channel in the ECs of the resistance artery. Activation of only three channels in a cluster of four was required to maximize vasodilating through the activation of intermediate, small, and calcium-sensitive potassium channels in ECs [71].

In addition, TRPV4 forms multimers with other molecules. TRP channels are a large superfamily of ion channels, including TRPV, TRPC, TRPP, etc. Studies showed that a heteromeric TRPV4-C1-P2 complex was formed in primary cultured rat mesenteric artery ECs and HEK293 cells co-transfected with TRPV4, TRPC1, and TRPP2. In functional experiments, the TRPV4-C1-P2 complex was sensitive to shear stress, mediated the generation of cation current, and was involved in regulating intracellular Ca²⁺ concentration and cell function. The pore inactivation mutants targeting these three TRP subtypes almost completely eliminated the cationic current and Ca²⁺ increase induced by fluid shear force. This indicated that all three TRP subunits were involved in the formation of ionic permeable channels [72].

While the function of TRPV4 as an endothelial mechanical sensor is well established, its impact on EndMT remains unclear. Existing research primarily focuses on the relationship between TRPV4 and epithelial-mesenchymal transition (EMT). EndMT is a special form of EMT. During the transformation process, both types of cells downregulate their original epithelial or endothelial characteristics to obtain mesenchymal characteristics [51, 73]. Knockdown of TRPV4 blocked EMT induced by matrix stiffness and transforming growth factor-beta 1 (TGF-β1) in mouse epidermal keratinocytes [74]. The migration ability of mouse dermal fibroblasts pretreated with TRPV4 antagonists was weakened. The expression of EMT markers [neural cadherin (N-cadherin), α-SMA] was downregulated. Under different matrix hardness conditions, the adhesion properties of cells pretreated with TRPV4 antagonists decreased [75]. Furthermore, studies confirmed that TRPV4 induced EMT in breast cancer and gastric cancer [76, 77]. Both EndMT and EMT were regulated by signaling pathways, including TGF-β/Smad and NOTCH. Both were related to vascular diseases like fibrosis and atherosclerosis [43]. Although the role of TRPV4 in EMT is relatively well-defined, its direct connection to EndMT requires further experimental validation.

#### ENaC

The epithelial sodium channel (ENaC) was present in vascular ECs. ENaC channels in ECs not only function in sodium ion transport but also have the ability to sense mechanical forces [78]. The research found that shear stress, tensile stress and hydrostatic pressure activated the ENaC channel in ECs [79, 80]. ENaC was composed of three subunits: α, β, and γ. The N-glycosylated asparagine existing in the palmar and phalangeal regions of αENaC is a key part of sensing shear stress. Glycosylated asparagine and its N-glycan, as potential connection points, enable ENaC to sense shear stress through interaction with the extracellular matrix [81]. Chronic inflammation-induced overactivation of ENaC led to a decrease in NO production in ECs, resulting in endothelial dysfunction and contributing to vascular diseases like atherosclerosis [82]. Another study also confirmed that the adaptive changes of ENaC in response to shear stress were associated with NO. ENaC in arteries increased vascular tension, while inhibiting ENaC led to vascular dilation and decreased tension. The vasodilating effect caused by the ENaC inhibitor amiloride disappeared after the removal of ECs. The vasodilating effect of the eNOS inhibitor l-NAME was consistent with that of amiloride. This indicated that ENaC regulated the carotid artery’s response to shear stress by influencing the eNOS/NO pathway [83] (Fig. 4a).

### G protein-coupled receptor (GPCR)

#### H1R

The H1 receptor (H1R) is a type of histamine receptor primarily distributed in various cells, including endothelial and smooth muscle cells [84]. The research suggested that mechanically sensitive H1R was a fluid shear stress sensor in ECs. In the fluorescence resonance energy transfer (FRET) experiment, the donors and recipients were attached to different positions of H1R, respectively, to monitor its conformational changes when stimulated. The results showed that when H1R was mechanically stimulated, the distance between the donor and the recipient near the C-terminal helix 8 (H8) was increased, resulting in a reduction of the FRET signal. The reduction amplitude of this FRET signal was significantly greater than the FRET signal changes caused by agonists like histamine at the maximum effective concentration. Those results indicated that mechanical stimulation and agonists induced different conformational changes of H1R. Further analysis indicated that the elongation of H8 was the key structural change in H1R in response to mechanical stimulation. This elongation enabled H1R to bind better with the G protein, thereby activating downstream signaling pathways, leading to an increase in intracellular calcium ion concentration and the production of NO, and further triggering vasodilation [85]. Therefore, the conformational changes of H1R when subjected to mechanical stimulation, especially the elongation of H8, are important bases for its function as a mechanically sensitive receptor.

#### GPR68

The activation of the G protein-coupled receptor 68 (GPR68) depends on the presence of protons and is most sensitive to the shear stress response within the pH range of 6.9–7.4. This indicates that GPR68 not only senses shear stress but also combines chemical stimulation, thereby triggering the dynamic response of cells to mechanical force and pH changes in their microenvironment [86]. GPR68 is a Gα_q/11_ coupled receptor. Studies have shown that GPR68 can respond to shear stress, thereby activating Gα_q/11_ signaling and increasing intracellular Ca^2+^ levels. In GPR68 knockout mice, the deletion of GPR68 disrupts the vasodilation response caused by increased blood flow. This further confirms that GPR68 mediates vasodilation and remodeling caused by blood flow. The mechanism by which GPR68 responds to shear stress has not been fully clarified, but it is known that it involves a phospholipase C (PLC)-dependent signaling pathway. When shear stress activates GPR68, it activates PLC and thereby increases the intracellular Ca^2+^ concentration [87].

#### B2R

Bradykinin 2 receptor (B2R) is specifically expressed on the cell membrane of vascular ECs [88, 89]. Studies found that fluid shear force and low osmotic stress led to a significant increase in the expression level of B2R in ECs. In response to fluid shear stress, B2R changed from an inactive to an active conformation. This indicated that B2R converted mechanical signals into biological signals. The conformational changes of B2R were blocked by B2-selective antagonists. When the fluid shear stress was removed, B2R returned to its original conformational state [90].

**Fig. 5:**
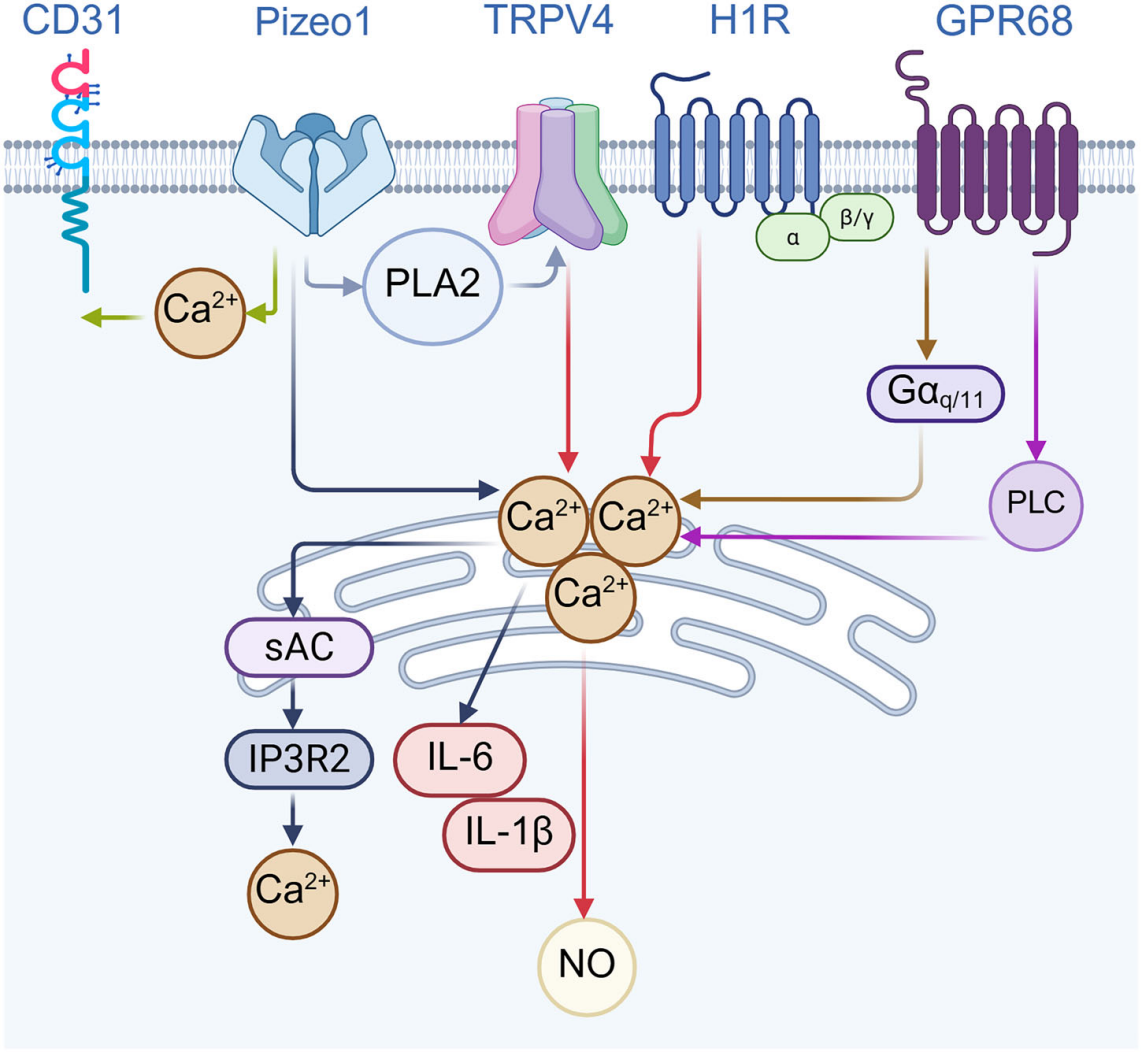
Ion channels and GPCR engage in a complex interaction mediated by Ca^2+^. This figure summarizes eight signaling pathways. (1) Piezo1-PLA2-TRPV4-Ca^2+^; (2) Piezo1-Ca^2+^-CD31; (3) Piezo1-Ca^2+^-sAC-IP3R2-Ca^2+^; (4) Piezo1-Ca²⁺-IL-6、IL-1β-EndMT; (5) TRPV4-Ca²⁺-NO; (6) H1R-G protein-Ca²⁺-NO; (7) GPR68-Gα_q/11_-Ca^2+^; (8) GPR68-PLC-Ca²⁺. (Created with BioRender.com).

As mechanical sensors, GPCR and ion channels interact with each other through calcium signaling. Among ion channels, Piezo1 activation stimulates PLA2, which in turn promotes the opening of the TRPV4 channel. This cascade results in a sustained increase in intracellular Ca²⁺ levels, ultimately triggering adherens junction disassembly and actin cytoskeleton remodeling [62]. Piezo1 and CD31 act synergistically to promote cell junction and cytoskeleton remodeling via Ca²⁺ influx [63]. Upon activation of Piezo1 by shear stress, Piezo1 amplifies calcium signals via sAC-mediated cAMP-sensitive IP3R2 to maintain ECs morphology and vascular function [65]. Furthermore, Piezo1 triggers Ca²⁺ influx and promotes the secretion of IL-6 and IL-1β, thereby mediating disturbed flow-induced endothelial–mesenchymal transition [66]. Under shear stress, TRPV4 undergoes distribution and conformational changes, promoting vasodilation through Ca²⁺ signaling [68, 69]. For GPCR, upon mechanical stimulation, the C-terminal H8 domain of the H1R undergoes elongation, which enhances its interaction with G proteins. This leads to an increase in intracellular Ca²⁺ concentration and NO production, ultimately inducing vasodilation [85]. GPR68 activates two signaling pathways (Gα_q/11_ and PLC) to elevate intracellular Ca²⁺ levels [87]. It is worth noting that ion channels and GPCR do not operate independently; instead, they can jointly regulate downstream signals and functions through Ca²⁺. They collectively regulate ECs responses to shear stress and intracellular calcium signals, thereby maintaining normal vascular physiological functions. Future studies could focus on the synergistic mechanism of calcium signaling among mechanical sensors under OSS, and clarify whether direct or indirect interactions exist between mechanical sensors and downstream signaling pathways (Fig. 4b) (Fig. 5).

### Others

#### CD31

CD31 (gene name: PECAM-1) is a transmembrane glycoprotein located at the intercellular junctions and is highly expressed in ECs. CD31 (directly transmitted mechanical force), vascular endothelial cadherin (VE-cadherin) (acted as a connector protein), and vascular endothelial growth factor receptor 2 (VEGFR2) (activated phosphatidylinositol-3-OH kinase) form a mechanical sensory complex to sense shear stress and conduct mechanical signals [91]. Daniel E. Conway et al. developed a FRET-based tension sensor to understand how the above-mentioned mechanical sensory complex sensed fluid shear stress and to monitor the tension changes of VE-cadherin and CD31 at the junctions of ECs in real-time. After the application of shear stress, FRET measurements showed that the VE-cadherin tension decreased rapidly, which was consistent with the decrease in the total tension at the connection. Meanwhile, blood flow led to an increase in CD31 tension at the junctions between bovine ECs, while there was no change at the non-junctions. Shear stress promoted the combination of CD31 and vimentin. The combination of the two was a necessary condition for CD31 to generate tension. The tension changes of VE-cadherin and CD31 were interdependent. That is, the tension changes of the two induced by shear stress required the existence of each other as a prerequisite. However, when vimentin expression was inhibited, the increase in tension of CD31 was blocked. While the tension of VE-cadherin was not affected. This indicated that the mechanical changes of the two were not completely synchronous or mutually restrictive. This discovery also revealed the dual functions of CD31 in the signal transduction of shear stress: it participated in signal transmission through mechanical conduction (dependent on tension), and also had a signal regulation mechanism independent of tension changes [92]. CD31 as a classic endothelial cell marker, is commonly used to verify the occurrence of EndMT. Studies have shown that OSS treatment significantly reduced CD31 expression in ECs [93–95]. The underlying mechanism may involve OSS-induced elevation of DNA methylation in the CD31 gene promoter region, which inhibits CD31 expression [95]. Conversely, other studies found that OSS upregulated CD31 to promote inflammation and atherosclerosis: OSS stabilized CD31 protein levels by downregulating the CDH1-dependent ubiquitin-proteasome degradation pathway, thereby facilitating endothelial inflammatory responses [96]. In ApoE^–/–^ mice, endothelial CD31 deficiency significantly reduced atherosclerotic plaque formation and local macrophage infiltration in the lesser curvature of the aortic arch (the major OSS-exposed region) [97]. The opposing expression patterns and effects of CD31 under OSS regulation may stem from differences in regulatory levels. At the synthesis level, OSS directly inhibits CD31 transcription via DNA methylation, thereby affecting endothelial phenotypic transition. At the degradation level, OSS upregulates CD31 protein levels by suppressing relevant degradation pathways.

#### Integrin

Integrins are heterodimer transmembrane glycoprotein receptors composed of α and β subunits. The response of ECs to the mechanical transduction process depends on the activation of integrin α5. Integrin α5 is only activated by OSS and not by continuous LSS [98]. Lipid rafts are membrane microdomains rich in cholesterol, sphingolipids, and various signal transduction molecules, mediating cellular signal transduction. OSS increases the level of activated integrin α5 in the lipid raft by regulating membrane cholesterol and fluidity. Silencing the lipid raft marker caveolin-1 inhibits the translocation and activation of integrin α5 induced by OSS. PCL surgery was performed in mice with low-density lipoprotein receptor deficiency. In the atherosclerotic plaques of the left blood flow disorder area, integrin α5 activation and ECs dysfunction were observed. Silencing integrin α5 significantly alleviated ECs dysfunction in this area. After treating HUVECs with OSS, OSS induced the translocation of integrin α5 to the lipid raft. This process was time-dependent. The use of integrin α5 neutralizing antibody dose-dependently inhibited the lysis of OSS-induced NOD-like receptor family pyrin domain containing 3 (NLRP3) inflammasome activation markers procaspase-1 and pro-IL-1β. This suggested that integrin α5 activation was necessary for NLRP3 inflammasome activation [99]. Furthermore, the deficiency of endothelin α5 significantly reduced the formation of atherosclerotic plaques [100].

#### ALK5

Activin receptor-like kinase 5 (ALK5) is a transmembrane protein belonging to the TGF-β receptor family, which directly senses and transduces mechanical signals as a mechanical sensor under shear stress. Shear stress mediates ALK5 expression to regulate ECs activation [101]. Some studies have shown that ALK5 activation depended on TGF-β signaling [102–104]. During embryonic heart valve development, shear stress activates the TGF-β/ALK5 signaling pathway to induce EndMT [105]. However, there is also an opposing view that the activation of ALK5 does not require TGF-β. The magnetic bead tension experiment found that after applying a mechanical force of 10 pN to the magnetic beads coated with anti-ALK5 antibody. The phosphorylation level of Smad2 in ECs was significantly increased. This effect still existed under serum-free conditions (excluding the interference of TGF-β) and after treatment with TGF-β neutralizing antibodies. This indicated that the activation of ALK5 did not depend on TGF-β. OSS activated the ALK5-Shc-Smad2 signaling pathway, thereby promoting the occurrence of EndMT. Meanwhile, the activation of ALK5 can also occur independently of other mechanical sensors (CD31, PLXND1). Because ALK5 still independently responded to shear stress in PECAM-1 knockout or PLXND1 knockout cells. The kinase inhibitor SB431542 completely blocked the activation of Smad2 induced by shear stress. This indicated that the kinase activity of ALK5 was at the core of mechanical signal transduction. The above research indicates that OSS directly activates ALK5. ALK5 possesses an independent mechanical sensing function [37].

#### Plexin D1 (PLXND1)

PLXND1 is a key Ca^2+^ -dependent cell surface receptor. When shear stress acted on ECs, it induced the formation of the PLXND1/neuropilin-1 (NRP1)/VEGFR2 mechanical complex. This mechanical complex regulates changes such as the cytoskeleton and cell adhesion. Knockout of PLXND1 led to a weakened response of ECs to shear stress, including the inhibition of phosphorylation of signal transducers AKT, extracellular signal-regulated kinase 1 and 2 (ERK1/2), and eNOS [106]. Under OSS conditions, PLXND1 up-regulated the expression of pro-inflammatory genes [C-C motif chemokine ligand 2 (CCL2), VCAM1], thereby inducing atherosclerosis. In ApoE^–/–^ mice, arterial plaques in ECs of PLXND1-deficient mice were significantly reduced [107].

#### BACH1

In atherosclerotic plaques of humans and mice, the expression of BTB and CNC homology 1 (BACH1) in their ECs was upregulated. BACH1 mediated the pro-inflammatory response of ECs caused by OSS. In HUVECs with BACH1 knockdown, the expression levels of endothelial adhesion molecules (ICAM1 and VCAM1) were decreased. In the BACH1 knockout mouse, atherosclerotic plaques were significantly improved. Meanwhile, the content of macrophages within the plaques was also decreased. Under the stimulation of OSS, BACH1 and Yes-associated protein (YAP) were induced and transferred to the nuclei of ECs. BACH1 upregulated YAP expression by binding to the YAP promoter. BACH1 formed a complex with YAP and induced the transcription of endothelial adhesion molecules. Overexpression of YAP in ECs counteracted the anti-atherosclerotic effect mediated by BACH1 deletion in mice. The above studies demonstrated that BACH1 sensed OSS and induced vascular inflammation and atherosclerosis through the BACH1-YAP transcription network [108]. Although the above-mentioned mechanical sensors are closely related to the adaptive changes of ECs induced by shear stress and vascular development, the regulatory mechanism of EndMT still requires experimental verification (Fig. 4c).

## Mechanism of the ONSET of EndMT induced by OSS

### TGF-β-Smad signaling pathway

#### FGFR1-TGF-β-Smad2

**Fig. 6:**
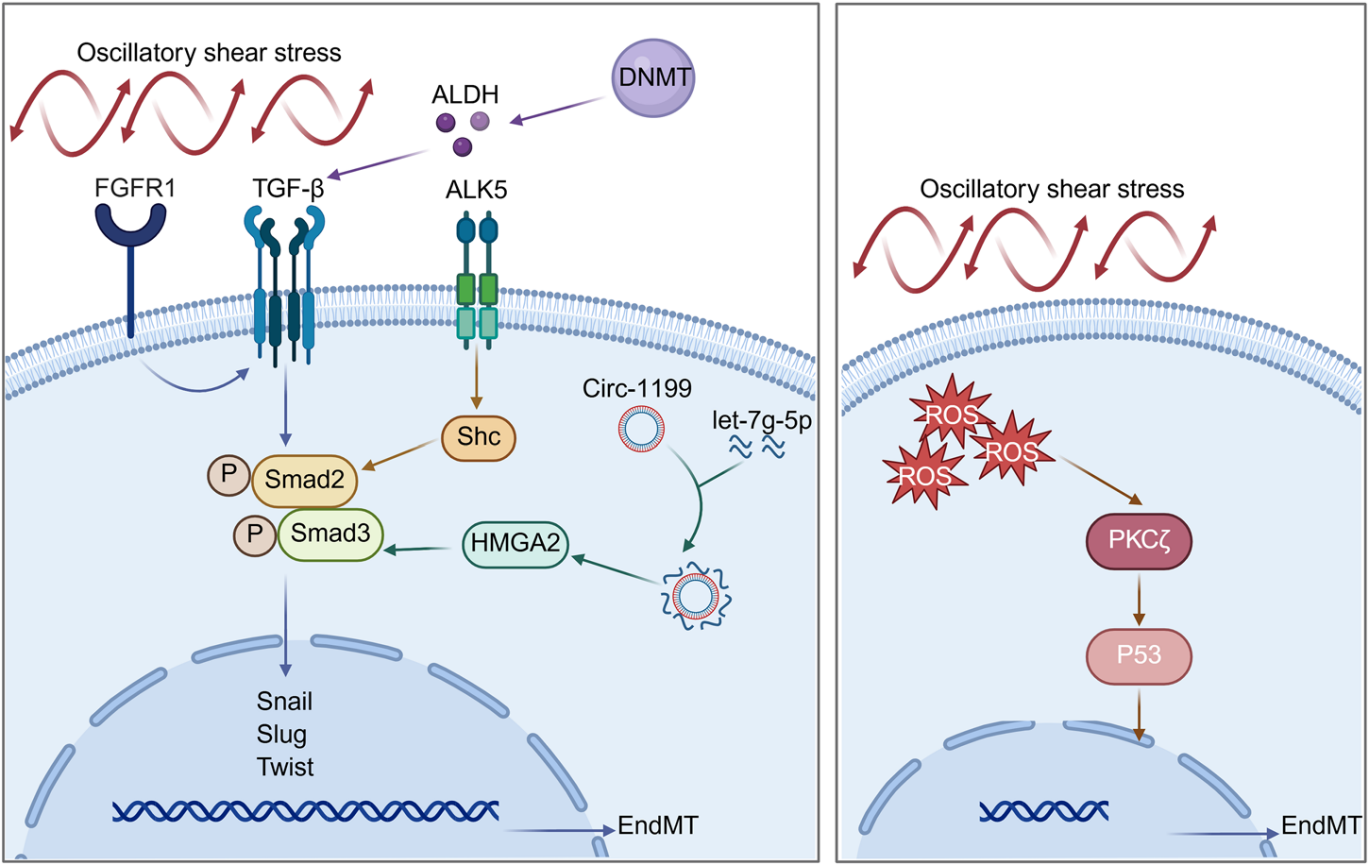
Signaling pathways of OSS-induced EndMT development. The TGF-β/Smad signaling pathway regulates EndMT through multiple mechanisms: (1) The deletion of FGFR1 activates TGF-β/Smad2 and promotes EndMT; (2) DNMT1 mediates ALDH methylation and activates the TGF-β pathway through Smad2/3 phosphorylation; (3) The mechanical force receptor ALK5 binds to Shc and directly transmits mechanical signals to activate Smad2; (4) Circ-1199 sponges let-7g-5p, releases HMGA2 to activate Smad3, and eventually activates the expression of key transcription factors Snail, Slug, and Twist in EndMT. In addition, OSS downregulates p53 through the ROS-PKCζ axis, promoting the occurrence of EndMT. (Created with BioRender.com).

Previous studies identified fibroblast growth factor receptor 1 (FGFR1) as a critical regulator of TGF-β-induced EndMT (Fig. 6, left) [109]. OSS reduced the expression of FGFR1 in HUVECs and activated TGF-β signaling to induce EndMT. FGFR1 knockout ApoE^–/–^ mice exhibited an increase in atherosclerotic plaque lesions in the aortic arch, root, thoracic aorta, and abdominal aorta. This knockout also activated TGF-β signaling and enhanced the expression of mesenchymal cell markers, specifically α-SMA and NOTCH3, leading to extensive deposition of collagen and fibronectin. These were all typical manifestations of EndMT. In a study involving patients with coronary artery disease, it was observed that FGFR1 expression in the endothelium of the left coronary artery decreased as the severity of the disease increased. However, the expressions of p-Smad2, EndMT markers (NOTCH3, SM22α), extracellular matrix proteins (collagen, fibronectin) and inflammation-related proteins (ICAM-1, VCAM-1) were gradually increased. Based on the above findings, inhibition of the EndMT process or restoration of endothelial FGF signaling pathways are expected to be effective strategies for the treatment of atherosclerosis [20].

### DNMT1-ALDH-TGF-β-Smad2/3

Epigenetic regulation mediated by DNA methylation is the key mechanism by which shear stress affects EndMT [95]. DNA methyltransferase (DNMT) maintains DNA methylation after DNA replication. The upregulation of endothelial DNMT1 by OSS promotes the methylation of the aldehyde dehydrogenase (ALDH) promoter and thereby inhibits the expression of ALDH. The dysregulation of ALDH2, ALDH3A1, and ALDH6A1 leads to impaired biosynthesis of β-alanine, carnitine, and acetyl-CoA, thereby increasing the phosphorylation of Smad2/3 and activating the TGF-β signaling pathway, and ultimately causing EndMT and atherosclerosis. 5-aza-2’-deoxycytidine (5-Aza) is a DNMT inhibitor that effectively inhibits EndMT induced by disordered blood flow. The results showed that 5-Aza significantly inhibited the expression of EndMT-related genes induced by OSS. Furthermore, pretreatment with 5-Aza inhibited the migration and proliferation of ECs and maintained the integrity of the junctions between ECs. In vivo experiments further confirmed that 5-Aza effectively inhibited EndMT. Studies showed that 5-Aza treatment significantly reduced the expression of ALDH2, ALDH3A1, and ALDH6A1. It also significantly reduced EndMT induced by disturbed blood flow, manifested as decreased expression of p-Smad2/3 and the mesenchymal marker SM22α [38] (Fig. 6, left).

### OSS-ALK5-Shc-Smad2

The ALK5-src homology and collagen (Shc) signaling pathway plays a pivotal role in regulating EndMT. ALK5, as a unique mechanical force receptor, is the main receptor mediating mechanical force-induced EndMT. When ALK5 is activated, its adapter protein Shc acts as a key downstream driver, participating in the transmission of mechanical force signals. Additionally, it leads ECs to express mesenchymal markers such as NOTCH3 and fibronectin, thereby promoting inflammatory responses and extracellular matrix remodeling, which ultimately exacerbates the formation of atherosclerotic plaques. Shc in ECs was specifically knocked out in ApoE^–/–^ mice, and a PCL model was constructed. The results showed that the aortic arch plaque area of Shc knockout ApoE^–/–^ mice was 70% smaller than that of Shc normally expressing ApoE^–/–^ mice. The proportion of p-Smad2-positive cells in the LCA endothelium was decreased. And the expressions of mesenchymal markers (NOTCH3, fibronectin, and α-SMA) were significantly decreased. ALK5-Shc signaling pathway is an important mechanism connecting OSS and EndMT. ALK5, as a unique mechanical sensor, can respond to mechanical forces and mediate the conduction of cell signals [37] (Fig. 6, left).

### Circ-1199-let-7g-5p-HMGA2-Smad3

The expression of circular RNA 1199 (Circ-1199) was significantly elevated in the exosomes released by endothelial progenitor cells after OSS treatment. Circ-1199 relieved the inhibitory effect of microRNA let-7g-5p (let-7g-5p) on high mobility group AT-hook 2 (HMGA2) by adsorbing let-7g-5p, thereby upregulating the expression of HMGA2. The increase of HMGA2 further activated the p-Smad3/Smad3 and Snail signaling pathways, promoting the transformation of endothelial progenitor cells into mesenchymal cells (Fig. 6, left). The dual-luciferase reporter gene assay verified the binding ability between Circ-1199 and let-7g-5p, as well as between let-7g-5p and HMGA2. Overexpression of Circ-1199 or knockdown of HMGA2 both up-regulated the expressions of Snail1, Twist, and Slug, thereby promoting EndMT [110].

### ROS-PKCζ-p53 signaling pathway

OSS induced EndMT in endothelial progenitor cells via the reactive oxygen species (ROS)-protein kinase C zeta (PKCζ)-p53 signaling axis (Fig. 6, right). The morphology of endothelial progenitor cells changed from pebble-like to spindle-shaped, and the intercellular gap increased. At the same time, the angiogenic capacity of endothelial progenitor cells in vitro was significantly decreased. Compared with LSS, the production of ROS induced by OSS increased by 3-5 times [95]. ROS acted as a rapid response mediator, and its accumulation increased rapidly within 5 min after OSS stimulation and reached its peak at 15 min. OSS reduced the expression of p53 in endothelial progenitor cells in a time-dependent manner. The p53 agonist tenovin-1 inhibited the expression of mesenchymal cell markers induced by OSS and relieved the inhibitory effect of OSS on the cell migration and angiogenic capacity of endothelial progenitor cells. Further studies found that OSS downregulated p53 by activating the ROS-PKCζ signaling pathway. OSS stimulation rapidly induced the accumulation of ROS and reached the peak at 15 min. Meanwhile, the level of p-PKCζ also increased in a time-dependent manner, and the increase was most significant when OSS acted for 10 min. Both the ROS remover N-acetylcysteine and the PKCζ inhibitor Go6983 effectively blocked the down-regulation of p53 induced by OSS. To verify the role of p53 in the formation of new intima after arterial injury, the authors conducted in vivo experiments. Lentiviral vector-mediated Tp53 overexpression in endothelial progenitor cells was transplanted into the left common carotid artery of rats to construct the PCL model. The formation of new endometrium in this group of rats was significantly reduced, and the expressions of α-SMA and SM22α in the endometrium decreased [111].

Although OSS triggers EndMT through ROS accumulation, the specific type of mechanical sensor involved in this process is not yet clear in current research. During the migration of polymorphonuclear leukocytes across the vascular endothelium, the Piezo1-nicotinamide adenine dinucleotide phosphate oxidase 4 (NOX4) signaling axis was activated by sensing mechanical tension, which generated ROS and thereby enhanced the bactericidal function [112]. In the heart, Piezo1 mediated Ca²⁺ influx to activate the Ras-related C3 botulinum toxin substrate 1 (Rac1)-nicotinamide adenine dinucleotide phosphate oxidase 2 (NOX2)-ROS-Ca²⁺ cascade reaction, regulated ROS homeostasis and maintained normal cardiac physiological functions [113]. Therefore, we make the assumption that Piezo1 may correspondingly participate in the ROS accumulation stimulated by OSS. However, this speculation requires further investigation.

Excessive ROS production promotes foam cell formation and exacerbates inflammatory responses. NO is a core molecule maintaining the integrity of vascular endothelial function. However, ROS rapidly reacts with NO to form peroxynitrite, leading to a significant reduction in NO bioavailability and endothelial dysfunction [114, 115]. Additionally, elevated ROS levels produced by NOX2-containing nicotinamide adenine dinucleotide phosphate (NADPH) oxidase independently induced endothelial dysfunction [116]. In the early stage of atherosclerosis, mitochondrial-derived ROS can mediate lysophosphatidylcholine-induced ECs activation, accelerating the initiation of atherosclerosis processes [117]. Sustainedly elevated ROS activates the inflammatory phenotype of ECs, providing a pathological basis for atherosclerosis [118, 119]. Beyond endothelial injury, ROS also induce the high expression of signal transducers and activators of transcription 5 (STAT5) in macrophages, promoting their preferential differentiation into foam cells [120]. Meanwhile, excessive ROS production in mitochondria facilitates the biosynthesis of proinflammatory cytokines [121], collectively driving the progression of atherosclerosis through the accumulation of foam cells and inflammation.

## Conclusion

Atherosclerosis is a prevalent and serious cardiovascular disease with a complex pathogenesis involving various factors. The promotion of EndMT by OSS plays a crucial role in the progression of atherosclerosis. This review summarized the effects of OSS on EndMT from three aspects: the phenomenon and mechanism of OSS-induced EndMT, the relationship between mechanical sensors and EndMT, and the atherosclerosis therapeutic strategies targeting EndMT. This review provides a theoretical basis for understanding and treating atherosclerosis from the perspective of OSS.

Apart from OSS, we must remain cognizant that, during the formation process of atherosclerosis and EndMT, the inflammatory response triggered by lipid accumulation is a crucial aspect. Inflammatory factors such as tumor necrosis factor-α (TNF-α), IL-1β, TGF-β, and Interferon-γ (IFN-γ) all induce endothelial dysfunction, thereby triggering EndMT [122, 123]. Among these, TNF-α and TGF-β have been widely used for constructing in vitro models of inflammation-induced EndMT. During inflammation-mediated EndMT, ECs also undergo a series of characteristic changes. Morphologically, ECs transform from a cobblestone-like phenotype to a spindle-shaped morphology. At the molecular level, the expression of endothelial markers (CD31, VE-cadherin) is downregulated. While mesenchymal markers (N-cadherin, Vimentin, α-SMA, Slug, Snail, TAGLN) are upregulated. Functionally, cell migration capacity is significantly enhanced [22, 55, 124–128]. Therefore, we can conclude that the basic characteristics of ECs undergoing EndMT caused by inflammation and OSS are consistent.

Meanwhile, inflammation and OSS induced EndMT share several commonalities: (1) Both drive ECs to transform from a regularly compact arrangement to an irregular mesenchymal morphology; (2) Both are accompanied by downregulation of endothelial markers and upregulation of mesenchymal markers; (3) Both enhance cell migration function; (4) Mechanistically, the TGF-β signaling pathway serves as a common key pathway for EndMT induction. Inflammatory mediators (TNF-α, IL-1β) and OSS all inhibit the expression of FGF receptors, thereby activating the TGF-β-Smad2/3 signaling pathway to initiate EndMT [20, 129]. Additionally, nuclear factor kappa B (NF-κB) is another core common signaling molecule [93, 130].

Notably, differences also exist between inflammation- and OSS-induced EndMT: (1) Phenomenologically, OSS, as a physical stimulus, primarily acts locally. It activates EndMT at arterial bifurcations or curved regions, thereby driving atherosclerotic plaque progression [131]. In contrast, inflammation, as a chemical signal, exhibits systemic characteristics. Chronic inflammation persistently activates systemic inflammatory responses, leading to multi-vessel involvement. Besides, inflammation-driven immune cell infiltration forms a complex immune microenvironment [132]. (2) Mechanistically, although NF-κB is a shared signaling molecule, its specific upstream and downstream molecules differ. Among inflammatory mediators, TNF-α and IL-6 mediate EndMT via the AKT/NF-κB pathway [133]. Yet another study demonstrates that TNF-α induces EndMT by activating the NF-κB/Snail signaling pathway through the α1 chain of collagen type VIII (COL8A1) [128]. In contrast, OSS triggers EndMT via the NOTCH1/p38 mitogen-activated protein kinase (p38 MAPK)/NF-κB signaling axis [93].

In atherosclerosis, ECs undergoing EndMT form a crosstalk network with smooth muscle cells and macrophages. Lineage tracing studies showed that at 8 and 30 weeks after high-fat diet feeding in ApoE^–/–^ mice, approximately 32.5% and 45.5% of mesenchymal cells in atherosclerotic plaques were derived from ECs, respectively [22]. ECs transform into α-SMA⁺ myofibroblast-like phenotypes via the TGF-β/Smad3 signaling pathway, directly participating in fibrous cap formation and disruption [134, 135]. Smooth muscle cell lineage tracing confirmed that approximately 20%-40% of α-SMA^+^ cells in atherosclerotic plaques were derived from non-smooth muscle cell sources [136]. The significant presence of CD31^+^ α-SMA^+^ cells in human coronary artery lesions indicated that EndMT is a crucial source of smooth muscle-like cells [135]. After ECs lineage tracing, mice were fed a high-fat western diet for 18 weeks until the advanced stage of atherosclerosis, and EndMT was observed in the brachiocephalic artery lesion areas. Data from male mice of this model showed that approximately 15% of α-SMA^+^ cells in the lesion fibrous caps originated from ECs rather than smooth muscle cells. In contrast, only 3% of α-SMA^+^ cells in female mice under the same dietary intervention were of ECs origin. This sex difference suggested that EndMT may be one of the important factors contributing to sex-specific differences in atherosclerotic development [137]. Single-cell RNA sequencing (scRNA-seq) also confirmed that ECs differentiate into pro-inflammatory EndMT subpopulations under OSS exposure, with transcriptomic overlap with smooth muscle cells and macrophages [93].

The chemotactic properties of macrophages are closely related to the EndMT induced by OSS. Macrophages with specific high expression of CD163 are defined as the CD163^+^ macrophage subset. Upon activation by the hemoglobin/haptoglobin complex, CD163^+^ macrophages secreted proinflammatory cytokines such as TNF-α and IL-6. These macrophages directly induced EndMT by upregulating the Snail transcription factor via the NF-κB pathway [23]. After myocardial infarction, macrophages promoted EndMT through membrane-type 1 matrix metalloproteinase (MT1-MMP)/TGF-β1 [138, 139]. These studies confirm that macrophages exert a driving effect on EndMT.

Bruton’s tyrosine kinase (BTK) is a key regulator of macrophage activation and polarization. In pulmonary vascular lesions, inhibition of BTK reduced the accumulation of M1 macrophages at the lesion site and further suppressed EndMT in pulmonary blood vessels [140]. Studies have verified that EndMT-induced cells significantly upregulated the expression of chemokines, including CCL2, CCL5, and monocyte chemoattractant protein-1 (MCP-1). These chemokines guided the directional migration of macrophages to the lesion site by binding to their corresponding receptors on the macrophage surface. Macrophages recruited to the lesion site further participated in inflammatory responses, and the regulation of ECs functions [124, 141]. In the liver fibrosis model, specific knockout of TGF-βRⅡ in sinusoidal endothelial cells not only directly inhibited EndMT but also reduced the recruitment of proinflammatory monocytes by downregulating chemokine expression [142].

Protein/histone acetylation plays a crucial role in the development of atherosclerosis [143–145]. This modification is regulated by histone acetyltransferases (HATs) and histone deacetylases (HDACs). OSS induces the sustained expression and nuclear accumulation of class I HDACs (HDAC1/2/3) and class II HDACs (HDAC5/7) via the phosphatidylinositol 3-kinase (PI3K)/AKT pathway. Specifically, OSS exacerbates endothelial oxidative stress through the HDAC1/2/3-nuclear factor erythroid 2-related factor 2 (NRF2)-NAD(P)H quinone dehydrogenase 1 (NQO1) pathway. It also promotes inflammation via the HDAC3/5/7-myocyte enhancer factor 2 (MEF2)-krüppel-like factor 2 (KLF2)-VCAM-1 pathway. These effects ultimately accelerate atherosclerosis progression [146]. Compared with healthy blood vessels, histone H3 lysine 9 (H3K9) and histone H3 lysine 27 (H3K27) acetylation levels are significantly increased in vascular smooth muscle cells and macrophages of advanced atherosclerotic lesions [147]. From a therapeutic perspective, inhibiting histone acetylation can suppress atherosclerosis. In vitro experiments confirmed that HDAC3 inhibition attenuated EndMT in HUVECs. In ApoE^–/–^ mice, HDAC3 expression was upregulated. The specific HDAC3 inhibitor RGFP966 inhibited EndMT, reduced lipid deposition, and alleviated atherosclerotic lesions [148]. However, other studies revealed a protective role of acetylation in atherosclerosis. Increased HDAC3 expression prevented atherosclerosis by inhibiting inflammation via the microRNA-19b/peroxisome proliferator-activated receptor γ (PPARγ)/NF-κB axis [149]. CD36 is a macrophage scavenger receptor that promotes the formation of macrophage-derived foam cells. Treatment with the histone deacetylase inhibitor trichostatin A (TSA) enhanced acetylation in the CD36 promoter region of macrophages. This elevation in acetylation led to upregulated CD36 expression, which in turn facilitated foam cell formation and accelerated atherosclerosis [150]. The bidirectional regulation of histone acetylation may be attributed to cell type specificity. Acetylation in the CD36 promoter region of macrophages promotes atherosclerosis by enhancing lipid uptake. In contrast, HDAC3 downregulation in ECs inhibits atherosclerosis by suppressing EndMT.

Inhibiting OSS-induced EndMT has emerged as a novel perspective for atherosclerosis treatment. Among clinical drugs, atorvastatin and metformin effectively alleviate OSS-induced EndMT [95, 151]. In laboratory studies, γ-secretase inhibitor RO4929097 [152], TGF-β1 receptor inhibitor SB431542 [153], and Smad2/3 inhibitor SB505124 [154] have all been shown to suppress OSS-induced EndMT.

Atherosclerosis-related EndMT exhibits a characteristic of multi-factor synergy. Therefore, current research on treating the disease by inhibiting EndMT not only focuses on OSS but also covers the exploration of mechanisms underlying EndMT induced by inflammation, lipids, and other factors. In terms of molecular targeted intervention: Adropin attenuates atherosclerosis by inhibiting TGF-β/Smad2/3 signaling pathway-mediated EndMT [155]; Epsin inhibitor API specifically suppresses the Epsin-FGFR1 interaction to inhibit EndMT [47]; Isoproterenol effectively inhibits EndMT via the adenylate cyclase 5 (AC5)-dependent cAMP/protein kinase a (PKA)/TGF-β axis [148]. Regarding traditional Chinese medicine, Dan-Shen-Yin effectively inhibits EndMT and reduces atherosclerotic plaque formation [156]. Dan-Shen-Yin comes from the Shi Fang Ge Kuo and is comprised of red sage (Salvia miltiorrhiza), sandalwood and fructus amomi (amomi fruit). The bioactive components in Salvia miltiorrhiza include tanshinone IIA [157], dihydrotanshinone I [158]and salvianolic acid A [159]. These bioactive components could efficiently treat atherosclerosis. It was reported that sandalwood extract exhibited anti-inflammatory activity and could attenuate hyperlipidemia in streptozotocin-induced diabetic rats [160, 161]. Fructus amomi has also been found to display anti-inflammatory properties [162, 163]. In nanoparticle systems, N-cadherin-targeted melanin nanoparticles reverse EndMT by downregulating the ras homolog family member a (RhoA)-dependent activation pathway [164]. Besides, Polydopamine nanoparticle-mediated mild photothermal therapy inhibited atherosclerotic plaque progression by regulating lipid metabolism in foam cells [165]. Macrophage biomimetic nanoparticles synergistically enhanced efferocytosis and cholesterol efflux to attenuate atherosclerotic progression [166]. Nanocarrier-mediated targeted delivery of anti-inflammatory cytokines reduced atherosclerosis in ApoE^–/–^ mice [167]. Nanoparticle-mediated delivery of pitavastatin inhibited atherosclerotic plaque destabilization/rupture in mice by regulating the recruitment of inflammatory monocytes [168].

However, we also noticed that many treatment strategies targeting EndMT still have some shortcomings. First, most studies are still at the cell and animal model stages. They still need to be confirmed by clinical research [156, 169]. Second, research on therapeutic strategies for multi-pathway collaborative regulation is still insufficient. Finally, how to enhance the specificity of drug treatment and reduce side effects is an urgent problem to be solved [170, 171].
